# A novel approach to thermographic images analysis of equine thoracolumbar region: the effect of effort and rider’s body weight on structural image complexity

**DOI:** 10.1186/s12917-021-02803-2

**Published:** 2021-03-02

**Authors:** Malgorzata Masko, Marta Borowska, Malgorzata Domino, Tomasz Jasinski, Lukasz Zdrojkowski, Zdzislaw Gajewski

**Affiliations:** 1 Department of Animal Breeding, Institute of Animal Science, Warsaw University of Life Sciences (WULS – SGGW), Nowoursynowska 100, 02-797 Warsaw, Poland; 2 Institute of Biomedical Engineering, Faculty of Mechanical Engineering, Białystok University of Technology, Wiejska 45C, 15-351 Bialystok, Poland; 3 Department of Large Animal Diseases and Clinic, Veterinary Research Centre and Center for Biomedical Research, Institute of Veterinary Medicine, Warsaw University of Life Sciences (WULS – SGGW), Nowoursynowska 100, 02-797 Warsaw, Poland

**Keywords:** Horse, Infrared thermography, Effort, Body weight, Temperature, Texture analysis

## Abstract

**Background:**

The horses’ backs are particularly exposed to overload and injuries due to direct contact with the saddle and the influence of e.g. the rider’s body weight. The maximal load for a horse’s back during riding has been suggested not to exceed 20% of the horses’ body weight. The common prevalence of back problems in riding horses prompted the popularization of thermography of the thoracolumbar region. However, the analysis methods of thermographic images used so far do not distinguish loaded horses with body weight varying between 10 and 20%.

**Results:**

The superficial body temperature (SBT) of the thoracolumbar region of the horse’s back was imaged using a non-contact thermographic camera before and after riding under riders with LBW (low body weight, 10%) and HBW (high body weight, 15%). Images were analyzed using six methods: five recent SBT analyses and the novel approach based on Gray Level Co-Occurrence Matrix (GLCM) and Gray Level Run Length Matrix (GLRLM). Temperatures of the horse’s thoracolumbar region were higher (*p* < 0.0001) after then before the training, and did not differ depending on the rider’s body weight (*p* > 0.05), regardless of used SBT analysis method. Effort-dependent differences (*p* < 0.05) were noted for six features of GLCM and GLRLM analysis. The values of selected GLCM and GLRLM features also differed (*p* < 0.05) between the LBW and HBW groups.

**Conclusion:**

The GLCM and GLRLM analyses allowed the differentiation of horses subjected to a load of 10 and 15% of their body weights while horseback riding in contrast to the previously used SBT analysis methods. Both types of analyzing methods allow to differentiation thermal images obtained before and after riding. The textural analysis, including selected features of GLCM or GLRLM, seems to be promising tools in considering the quantitative assessment of thermographic images of horses’ thoracolumbar region.

## Background

The measurement of superficial body temperature (SBT) via thermography is a common method used to monitor horses’ health status [1–3]. It allows for quantification of radiant energy emitted by the body surface since the body temperature is above absolute zero [4]. Alterations of emitted radiated power, which are proportional to the fourth power of the surface temperature [5], were widely used as a diagnostic tool in equine veterinary medicine [1, 2, 6, 7]. Diagnosed SBT increases pointed to an active inflammatory process and/or alterations in local blood flow corresponding to overload or injuries of underlying tissues [1, 3, 8].

The horses’ thoracolumbar region is particularly exposed to overload and injuries due to direct contact with the saddle and the influence e.g., of the rider’s body weight. Visser et al. (2014) demonstrated back problems, using horses’ responses to back palpation, in 31% of 2956 examined horses working in leisure or riding schools, participating in the competition, and housing at the stud farms [9]. However, in Haussler’s (1999) report, the prevalence of back disorders in horses ranged from 1 to 94% [10]. Also, Haussler and Jeffcott (2014) suggested an association between the type of back pain and the type of horse’s work [11]. Acute sacroiliac strain or subluxation was more prevalent in horses jumping at speed, whereas impinged or over-riding dorsal spinous processes were most common in showjumpers. Sacroiliac pain is common in dressage horses and causes impaired performance, usually without lameness. High incidence of sacroiliac and hindquarters problems are also shown in standardbred harness. The horse’s back problems resulting from long periods of extreme exercise and saddle-induced injuries are common in endurance [11]. Whereas in leisure and school horses, the incidence of soft tissue injuries usually results from the work with many different riders, often beginners, still learning how to sit properly [12, 13]. Moreover, in Visser et al.’s (2014) study, leisure and school horses were twice as often affected with back pain then other working horses [9].

The common prevalence of back problems in riding horses prompted the popularization of thermography of the thoracolumbar region [2, 6, 12, 14–16]. These measurements of SBT were used in horses to identify and localize spine-related diseases [2], to diagnose thoracolumbar lesions in equine athletes [6, 15], to evaluate a normal thermal pattern [14, 16], as well as to describe interactions between horse and rider [12]. Each of the cited studies has applied a different analysis method to interpret the superficial body temperature of the thoracic region of a horse’s back e.g., looking for thermographically portrayed “hot spots” or “cold regions” [2, 6], analyzing the range of temperatures for the back measured along three horizontal lines [14], comparing the average temperatures measured in three areas separated from the back surface [15], measuring the average temperature in the selected quadrate of the thermographic image from the thoracic region of the horse’s back [16] or evaluating the heat pattern of the thoracolumbar area using 37 reference points grouped into 7 regions of interest [12]. In the absence of a ‘gold standard’ for equine back thermal imaging, the detailed analysis of gathered thermographic images became a challenge. In all these recent studies, infrared radiation was presented as a thermogram, where the color gradient corresponds to the distribution of surface temperatures [4].

Since the thermogram remains an image, the computer-aided analysis of image texture such as Gray-Level Matrices (GLM) can be introduced [17]. GLM represents a group of non-linear texture operators which are statistics recording distribution and relationship of images pixels. This group mainly consists of three detailed approaches: Gray Level Co-Occurrence Matrix (GLCM), Gray Level Run Length Matrix (GLRLM), and Gray Level Size Zone Matrix (GLSZM) [17], among which GLCM and GLRLM application into thermal images analysis seem to be of particular interest. GLCM was successfully applied to the analysis of biogenic sedimentary structures [18] and rapid and constant monitoring of the hygienic condition of surfaces in the food industry [19]. This first GLM approach represented a collection of operators mapping image function to binary output [17]. On the other hand, GLRLM is a widely used method for extracting statistical features for medical images, e.g., ultrasound medical images [20] or magnetic resonance images [21]. This second GLM approach counts the number of aligned pixels with equal gray levels [17]. Both approaches were also applied together in other medical applications such as an analyzing radiographic images during the healing process [22] or histopathology images in a case of brain cancer diagnosis [23]. Due to numerous recent medical applications, the calculation of selected features of GLCM and GLRLM descriptors is presented here as an example of how indices of structural complexity can be used in the analysis of thermographic images of a horse’s back.

The study aimed at evaluating an advanced texture analysis technique for thermographic images of the thoracolumbar region of horses. In this novel application of the analysis method, the following textural parameters are proposed: Gray Level Co-Occurrence Matrix (GLCM) and Gray Level Run Length Matrix (GLRLM). Additionally, we studied if the superficial temperatures and the image texture of the back of leisure horses changed depending on effort and the rider’s body weight. For this purpose, results of five recent SBT analysis methods and the new computer-aided texture analysis method of the same thermographic images were obtained before and after riding, and with riders with lower or higher body weight.

## Results

**Fig. 1:**
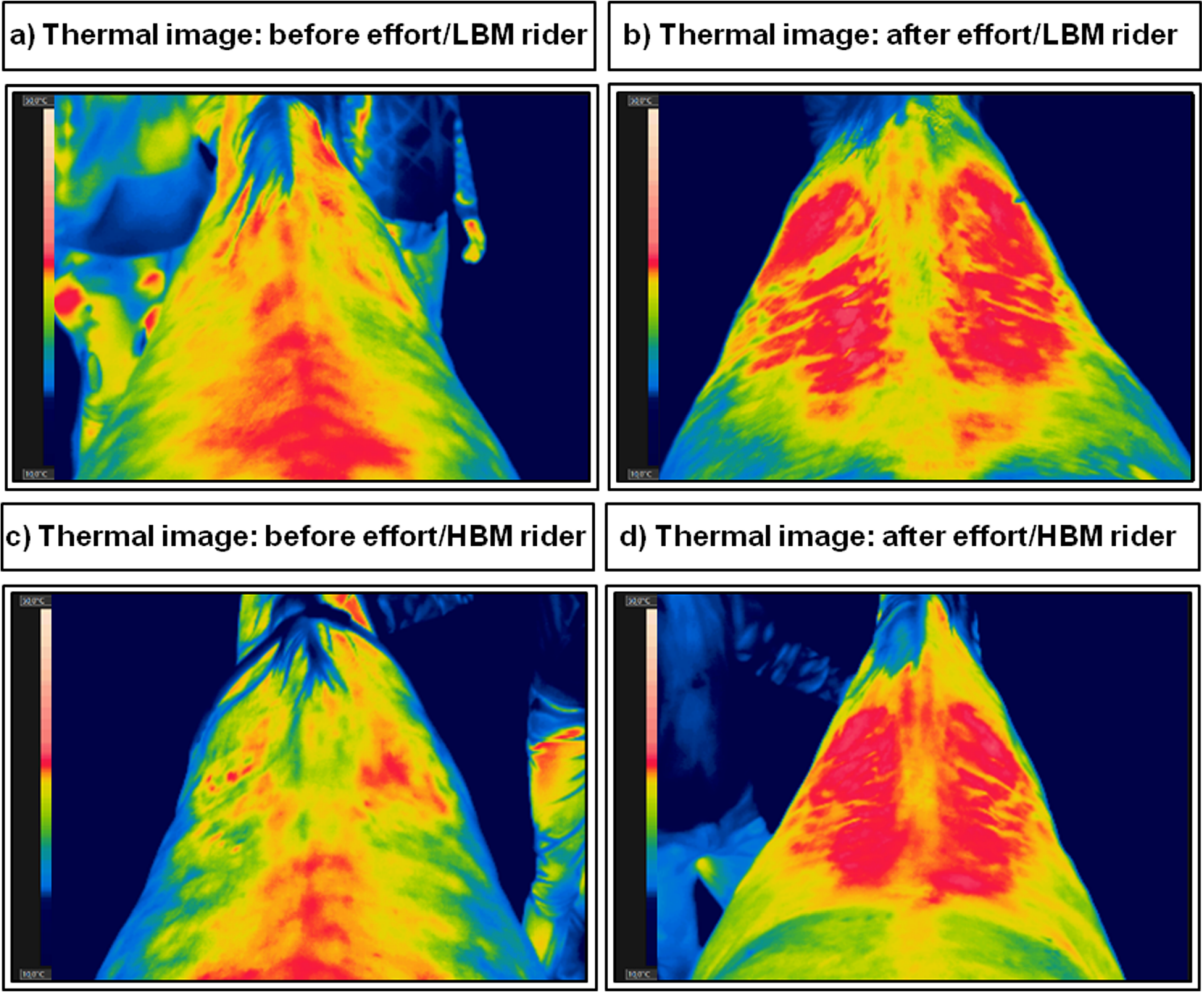
Samples of thermographic images of the thoracolumbar region of the same horse taken on two consecutive days: **a** before effort under LBW rider; **b** after effort under LBW rider; **c** before effort under HBW rider; **d** after effort under HBW rider

Sample thermographic images of the thoracolumbar region of the same horse taken on two consecutive days of the study were presented in Fig. 1.

Temperatures of the horse’s back obtained using method I were summarized in Table 1. The average temperatures (T_aver_) of the normal thermal profile of the horse’s back were higher after then before training session (*p* < 0.0001). The alterations in the thermal pattern, both “hot spots” and “cold region” have not been recognized, therefore the temperature values for the maximal temperature (T_max_) of alterations and the minimal temperature (T_min_) of alterations were not given. All detected temperature differences were in the range of 0.5 °C to 1 °C, therefore they were considered normal. There were no differences between the temperatures obtained for LBW (low body weight) and HBW (high body weight) groups, both before (*p* = 0.4697) and after (*p* = 0.5320) training session.

**Table 1:** Temperatures (°C) of the horse’s back obtained before and after a training session the use of method I [2, 6] for two groups of riders: low body weight (LBW) and high body weight (HBW)

	T_**aver**_ of NTP		T_**max**_ of alterations		T_**min**_ of alterations	
	LBW	HBW	LBW	HBW	LBW	HBW
Before	25.3 ± 1.33^a^	25.6 ± 1.21^a^	–	–	–	–
After	30.2 ± 1.88^b^	32.0 ± 2.12^b^	–	–	–	–
*p*	< 0.0001	< 0.0001	–	–	–	–

*T*_*aver*_ *of NTP* the average temperature of the normal thermal profile, *T*_*max*_ *of alterations* the maximal temperature of “hot spot”, *T*_*min*_ *of alterations* the minimal temperature of “cold region”. ^a, b^ - subsequent letters in superscript indicated differences before/after a training session and LBW/HBW. Additionally, differences before/after a training session indicated with the *p*-value. The significance level was established as *p* < 0.05

Temperatures of the horse’s back obtained using method II were summarized in Table 2. The differences between the temperature at midline and at subsequent positions (T_diff_) were lower after then before training session for all examined positions (the *p*-value range from *p* = 0.0441 to *p* < 0.0001). There were no differences between the temperatures obtained for LBW and HBW groups, both before (*p* > 0.05) and after (*p* > 0.05) training session, also for all examined positions.

**Table 2:** Temperatures (°C) of the horse’s back obtained before and after a training session with the use of method II [14] for two groups of riders: low body weight (LBW) and high body weight (HBW)

	**T**_**diff**_ **at position 0**		**T**_**diff**_ **at position 20**		**T**_**diff**_ **at position 40**	
	LBW	HBW	LBW	HBW	LBW	HBW
Before	−3.0 ± 0.12^a^	− 3.2 ± 0.09^a^	−1.2 ± 0.10^a^	− 1.4 ± 0.22^a^	− 1.0 ± 0.09^a^	− 1.0 ± 0.11^a^
After	− 1.2 ± 0.33^b^	− 1.0 ± 0.50^b^	− 0.95 ± 0.14^b^	− 1.01 ± 0.09^b^	− 0.6 ± 0.15^b^	− 0.8 ± 0.07^b^
*p*	< 0.0001	< 0.0001	0.0003	0.0025	0.0121	0.0381
	**T**_**diff**_ **at position 60**		**T**_**diff**_ **at position 80**		**T**_**diff**_ **at position 100**	
	LBW	HBW	LBW	HBW	LBW	HBW
Before	−1.0 ± 0.11^a^	− 1.1 ± 0.11^a^	− 1.2 ± 0.19^a^	− 1.1 ± 0.11^a^	−3.2 ± 0.21^a^	− 3.2 ± 0.15^a^
After	−0.6 ± 0.11^b^	−0.8 ± 0.09^b^	−0.90 ± 0.07^b^	− 0.90 ± 0.05^b^	− 1.3 ± 0.29^b^	−0.90 ± 0.62^b^
*p*	0.0021	0.0441	0.0187	0.0433	< 0.0001	< 0.0001

T_diff_ at positions 0, 20, 40, 60, 80, 100 - the differences between temperature at midline (0) and at subsequent positions: 0, 20, 40, 60, 80, 100. ^a, b^ - subsequent letters in superscript indicated differences before/after a training session and LBW/HBW. Additionally, differences before/after a training session indicated with the *p*-value. The significance level was established as *p* < 0.05

Temperatures of the horse’s back obtained using method III were summarized in Table 3. The average temperature of the left side of the muscles, thoracic vertebrae and right side of the muscles were always higher after effort then before (*p* < 0.0001). No differences between the temperatures obtained for LBW and HBW groups were noted (*p* > 0.05), also for all three features both before and after a training session.

**Table 3:** Temperatures (°C) of the horse’s back obtained before and after a training session with the use of method III [15] for two groups of riders: low body weight (LBW) and high body weight (HBW)

	T_**aver**_ of ML		T_**aver**_ of Th		T_**aver**_ of MR	
	LBW	HBW	LBW	HBW	LBW	HBW
Before	26.3 ± 1.10^a^	26.0 ± 1.13^a^	29.2 ± 1.60^a^	30.1 ± 1.55^a^	26.6 ± 1.10^a^	26.3 ± 0.90^a^
After	34.1 ± 1.70^b^	33.7 ± 1.29^b^	33.6 ± 1.22^b^	34.0 ± 0.95^b^	33.2 ± 1.43^b^	33.9 ± 1.04^b^
*p*	< 0.0001	< 0.0001	< 0.0001	< 0.0001	< 0.0001	< 0.0001

*T*_*aver*_ *of ML* the average temperature of left side of the muscles, *T*_*aver*_ *of Th* the average temperature of thoracic vertebrae, *T*_*aver*_ *of MR* the average temperature of right side of the muscles. ^a, b^ - subsequent letters in superscript indicated differences before/after a training session and LBW/HBW. Additionally, differences before/after a training session indicated with the *p*-value. The significance level was established as *p* < 0.05

Temperatures of the horse’s back obtained using method IV were summarized in Table 4. The average temperatures of the left side of the thoracic region (ArL) and the right side of the thoracic region (ArR) were also higher after effort then before a training session (*p* < 0.0001). As in previous measurements, there were no differences between the temperatures obtained for LBW and HBW groups, both before (ArL *p* = 0.7788; ArR *p* = 0.4197) and after (ArL *p* = 0.6326; ArR *p* = 0.4402) training session.

**Table 4:** Temperatures (°C) of the horse’s back obtained before and after a training session with the use of method IV [16] for two groups of riders: low body weight (LBW) and high body weight (HBW)

	T_**aver**_ of ArL		T_**max**_ of ArR	
	LBW	HBW	LBW	HBW
Before	28.2 ± 0.87^a^	27.9 ± 1.01^a^	28.0 ± 1.11^a^	28.3 ± 1.17^a^
After	33.2 ± 1.20^b^	34.9 ± 1.61^b^	33.0 ± 1.41^b^	35.1 ± 2.02^b^
p	< 0.0001	< 0.0001	< 0.0001	< 0.0001

*T*_*aver*_ *of ArL* the average temperature of left side of the thoracic area, *T*_*aver*_ *of ArR* the average temperature of right side of thoracic area. ^a, b^ - subsequent letters in superscript indicated differences before/after a training session and LBM/HBM. Additionally, differences before/after a training session indicated with the *p*-value. The significance level was established as *p* < 0.05

Temperatures of the horse’s back obtained using method V were constantly summarized in Table 5. The maximal temperatures of regions of interest 1–7, as well as of the entire body, were higher after a training session (*p* < 0.0001) in comparison to the resting stage. Once more, no differences (*p* > 0.05) between the temperatures obtained for LBW and HBW groups were noted, for all region of interests (ROIs) and entire bodies, alike before and after effort.

**Table 5:** Temperatures (°C) of the horse’s back obtained before and after a training session with the use of method V [12] for two groups of riders: low body weight (LBW) and high body weight (HBW)

	**T**_**max**_ **in ROI 1**		**T**_**max**_ **in ROI 2**		**T**_**max**_ **in ROI 3**		**T**_**max**_ **in ROI 4**	
	LBW	HBW	LBW	HBW	LBW	HBW	LBW	HBW
Before	31.4 ± 1.32^a^	31.6 ± 1.20^a^	31.2 ± 1.40^a^	31.4 ± 0.95^a^	31.1 ± 1.50^a^	31.3 ± 1.04^a^	31.0 ± 1.40^a^	31.0 ± 1.22^a^
After	33.5 ± 1.10^b^	33.7 ± 1.21^b^	33.6 ± 1.12^b^	33.8 ± 1.25^b^	33.7 ± 1.01^b^	33.8 ± 1.40^b^	34.4 ± 1.01^b^	34.3 ± 0.98^b^
*p*	< 0.0001	< 0.0001	< 0.0001	< 0.0001	< 0.0001	< 0.0001	< 0.0001	< 0.0001
	**T**_**max**_ **in ROI 5**		**T**_**max**_ **in ROI 6**		**T**_**max**_ **in ROI 7**		**T**_**max**_ **of entire bodies**	
	LBW	HBW	LBW	HBW	LBW	HBW	LBW	HBW
Before	31.2 ± 0.99^a^	31.0 ± 1.40^a^	31.1 ± 1.45^a^	30.8 ± 1.33^a^	30.9 ± 1.02^a^	30.7 ± 1.30^a^	31.2 ± 1.12^a^	30.9 ± 1.01^a^
After	34.4 ± 1.14^b^	34.5 ± 1.51^b^	33.2 ± 1.14^b^	33.4 ± 1.14^b^	33.3 ± 1.22^b^	34.5 ± 1.62^b^	33.5 ± 1.14^b^	33.8 ± 1.60^b^
*p*	< 0.0001	< 0.0001	< 0.0001	< 0.0001	< 0.0001	< 0.0001	< 0.0001	< 0.0001

*ROI* region of interest, *T*_*max*_ *of ROI 1–7* the maximal temperature of regions of interest 1–7, *T*_*max*_ *of entire body* the maximal temperature of all 7 ROIs. ^a, b^ - subsequent letters in superscript indicated differences before/after a training session and LBW/HBW. Additionally, differences before/after a training session indicated with the *p*-value. The significance level was established as *p* < 0.05

The features of the novel texture analysis methods were summarized in Table 6. Considering the GLCM, the lower Contrast (LBW *p* < 0.0001; HBW *p* = 0.0010), Entropy (LBW *p* = 0.0050; HBW *p* = 0.0108), DifVarnc (difference variance; LBW *p* < 0.0001; HBW *p* = 0.0006) and DifEntrp (difference entropy; LBW *p* < 0.0001; HBW *p* = 0.0006) were noted after then before training session, in both LBW and HBW groups. Similarly, the lower (*p* = 0.0470) Correlate was calculated after then before effort however only in the HBW group. The higher InvDefMom (inverse different moment; LBW *p* = 0.0001; HBW *p* = 0.0073) after then before effort were noted also in both LBW and HBW groups. Moreover, after training session the differences between LBW and HBW groups were observed when Contrast (*p* = 0.0015), Correlate (correlation; *p* = 0.0006), InvDefMom (*p* = 0.0010), Entropy (*p* = 0.0440), DifVarnc (*p* = 0.0001) and DifEntrp (*p* = 0.0001) were considered.

**Table 6:** Gray Level Co-Occurrence Matrix (GLCM) and Gray Level Run Length Matrix (GLRLM) of the horse’s back obtained before and after a training session with the use of method VI (novel texture analysis) for two groups of riders: low body weight (LBW) and high body weight (HBW)

**GLCM**	**Contras**		**Correlate**		**InvDefMom**		**Entropy**		**DifVarnc**		**DifEntrp**	
	LBW	HBW	LBW	HBW	LBW	HBW	LBW	HBW	LBW	HBW	LBW	HBW
Before	1.11 ± 0.49^a^	1.27 ± 0.46^a^	0.88 ± 0.09^a^	0.91 ± 0.06^a^	0.73 ± 0.07^a^	0.72 ± 0.05^a^	1.28 ± 0.30^a^	1.35 ± 0.22^a^	0.69 ± 0.27^a^	0.79 ± 0.26^a^	0.45 ± 0.08^a^	0.47 ± 0.06^a^
After	**0.46 ± 0.11**^**b**^	**0.78 ± 0.36**^**c**^	**0.92 ± 0.02**^**a**^	**0.87 ± 0.04**^**b**^	**0.82 ± 0.04**^**b**^	**0.77 ± 0.05**^**c**^	**1.07 ± 0.17**^**b**^	**1.19 ± 0.17**^**c**^	**0.31 ± 0.06**^**b**^	**0.49 ± 0.21**^**c**^	**0.32 ± 0.04**^**b**^	**0.40 ± 0.06**^**c**^
*p*	< 0.0001	0.0010	0.5140	0.0470	0.0001	0.0073	0.0050	0.0108	< 0.0001	0.0006	< 0.0001	0.0006
**GLRLM**	**RLN**		**GLN**		**LRE**		**SRE**		**Fraction**		**MRLN**	
	LBW	HBW	LBW	HBW	LBW	HBW	LBW	HBW	LBW	HBW	LBW	HBW
Before	747 ± 378^a^	896 ± 314^a^	793 ± 184^a^	916 ± 379^a^	177 ± 205^a^	133 ± 85^a^	0.24 ± 0.07^a^	0.26 ± 0.06^a^	0.16 ± 0.05^a^	0.16 ± 0.04^a^	0.11 ± 0.03^a^	0.12 ± 0.02^a^
After	**263 ± 101**^**b**^	**568 ± 327**^**c**^	**693 ± 171**^**b**^	**997 ± 421**^**a**^	**317 ± 279**^**b**^	**161 ± 111**^**c**^	**0.13 ± 0.02**^**b**^	**0.19 ± 0.05**^**c**^	**0.10 ± 0.02**^**b**^	**0.14 ± 0.03**^**c**^	**0.07 ± 0.01**^**b**^	**0.09 ± 0.02**^**c**^
*p*	< 0.0001	0.0043	0.0087	0.6218	0.0011	0.0002	0.0219	0.0008	0.0002	0.0219	< 0.0001	0.0004

*Contras* contrast, *Correlate* correlation, *InvDefMom* inverse different moment, *Entropy* entropy, *DifVarnc* difference variance, *DifEntrp* difference entropy, *RLN* run length nonuniformity, *GLN* Gray level non-uniformity, *LRE* long run emphasis, *SRE* short run emphasis, *Fraction* fraction of image in runs, *MRLN* run length nonuniformity moment. ^a, b^ - subsequent letters in superscript indicated differences before/after a training session and LBW/HBW. Additionally, differences before/after a training session indicated with the *p*-value, and LBW/HBW emphasized with bold font. The significance level was established as *p* < 0.05

Finally, the GLRLM analysis shown the lower RLN (run-length nonuniformity; LBW *p* < 0.0001; HBW *p* = 0.0043), SRE (short-run emphasis; LBW *p* = 0.0219; HBW *p* = 0.0008), Fraction (a fraction of image in runs; LBW *p* = 0.0002; HBW *p* = 0.0219) and MRLN (run-length nonuniformity moment; LBW *p* < 0.0001; HBW *p* = 0.0004) after then before training in both groups. Also, the lower GLN (Gray level non-uniformity; *p* = 0.0087) after then before training was noted in LBW group. The higher LRE (long-run emphasis; LBW *p* = 0.0011; HBW *p* = 0.0002) after then before effort were shown also in both groups. In a case of GLRLM, such features as RLN (*p* = 0.0003), GLN (*p* = 0.0037), LRE (*p* = 0.0052), SRE (*p* = 0.0010), Fraction (*p* = 0.0007), and MRLN (*p* < 0.0001) differed between LBW and HBW groups, however, also only after effort.

## Discussion

Effort-dependent differences in thermographic images of the thoracolumbar region were well determined using all applied classical thermographic analysis as well as a novel structural image complexity approach. Significant increases in T_aver_ of NTP (method I), T_diff_ at position 0–100 (method II), T_aver_ of ML, Th and MR (method III), T_aver_ of ArL and T_max_ of ArR (method IV), and T_max_ in ROI 1–7 (method V) were observed each time after exercise in comparison to horses back image at rest. Measuring the superficial body temperature, regardless of the used method, is still deemed to be the best method to demonstrate the changes that occur during physical exercise. During a training session, metabolic heat production increases as exercise intensity increases [24]. Only 20 to 25% of the energy used by a muscle is converted to mechanical energy, the remaining 75 to 80% is dissipated as heat [25]. Both, exercise intensity and the size of a muscle unit in a place of imaging are determinants of the rate of heat production. Therefore, the comparison of the effort depended differences in thermographic images may be conducted only on the same thermographic images. In this case, the subsequent analyzing methods are able to describe in a different way the same muscle units. Interestingly, the changes in back region activity may also be described by the new texture analysis approach. In the same experimental protocol, all GLCM and GLRLM features presented in Table 6. differed depending on effort, however some values increased (InvDefMom) while others decreased (Contras, Entropy, DifVarnc, DifEntrp, RLN, SRE, Fraction, MRLN). Changes of the other features (Correlate, GLN, LRE) seem to depend not only on effort, but also the rider’s body weight. Noteworthy results of our study suggest the selected features of GLCM and GLRLM analysis may also be used for a detailed evaluation of the level of heat production during physical exercise.

Since the rider’s body weight was reported as a major factor that influencing physical exercise in horses [26], the effective rider’s related horse load analysis approach has been requested. Powell et al. (2008) subjected the horses to a submaximal mounted standard exercise test under four conditions: carrying 15, 20, 25, or 30% of their body weight. They stated that horses carrying 10–15% of their body weight (about 50–75 kg of rider’s body weight) demonstrate no physiological changes [27]. Then the maximal load for a horse has been suggested to not exceed 20% of horses body weight (about 100 kg of rider’s body weight) [28, 29], seeing that exceeding load constituting 25% of horses body weight (about 125 kg of rider’s body weight) results in the basic physiological parameters increasing and post-exercise muscle pain [27, 28]. Wilk et al. (2020) applied thermographic imaging to determining differences in SBT distribution between horses ridden by two riders with varied body weight (rider’s body weight with saddle (kg)/% body weight of horse: LBW rider 50 kg/10.6%; HBW rider 100 kg/21.3%). The Authors demonstrated no differences in average SBT of the middle part of trunk (thoracolumbar region) between LBW and HBW rider’s tests [29]. Also Soroko et al. (2019) reported no influence of the rider’s weight on the saddle thermal pattern distribution, however these authors did not report the percent load on the horse’s body weight. In this publication, the load on the horse’s back was between 54.7 kg and 61.6 kg [30], although in the absence of data on the horse’s body weight it can only be assumed that it was about 10–15% of the horse’s body weight. In the study presented here, also no differences in SBT of the thoracolumbar region between LBW (average load 54.6 kg/10.1%) and HBW (average load 82.7 kg/15.3%) groups were observed. It is worth noting that the effect of the rider’s body weight was detectable using the structural image complexity analyses. In Gray Level Co-Occurrence Matrix analysis, values of Contras, Entropy, DifVarnc, and DifEntrp increased with increasing load, whereas values of Correlate and InvDefMom decreased. Also in Gray Level Run Length Matrix analysis, values of RLN, GLN, SRE, Fraction, and MRLN increased with increasing load, while LRE decreased. The relative higher Entropy and DifEntrp after working under the heavier rider may suggest a greater degree of energy dissipation. Also, other features of GLCM analysis, such as low Contrast, high InvDefMom, and low DifVarnc in the LBW group after effort, may indicate less variability in the structure of thermograms. Similarly, values of features of GLRLM analysis in the LBW group after effort, low RLN, low GLN, low SRE, low Fraction, and low MRLN may indicate less variability in the structure of thermograms. In the future study, determining the relationship between the GLM analysis results and the myoelectric activity of the underlying muscles may answer the question of whether the observed changes in the structure of the image are related to disorder work of the back muscles in response to higher body weight or better coordination or favorable work conditions of back’s muscle units in response to lower body weight. Further investigations of the utility of the texture analysis methods of thermographic images concerning the type of horse’s back-pain problem are also requested.0.

The main limitation of this study is that thermographic imaging cannot distinguish among the effects of the rider, the saddle fit, and the movements of the horse. The obtained results may only be evaluated concerning the experience design. Since the forces acting on the horse’s back vary depending on the saddle fit, rider’s training level, and rider’s body weight [31], all those factors should be included. Especially since both Meschan et al. (2007) and Belock et al. (2012) indicated that pressure is more concentrated with poorly fitted saddles with heavier riders [32, 33]. In a study presented here, all saddles were fitted properly and all riders demonstrated the same level of skills, therefore, we may suspect, that the texture analysis allows visualizing the superficial changes in response to the rider’s body weight. Seeing that, the rider’s body weight and saddle mass influenced the overall extension of the horse’s back while riding [34], it should be distributed as well as possible. Therefore, further investigation is needed to evaluate the direct load distribution over the horse’s back region concerning changes in the thermographic image texture. Such research can be carried by measured the force applied on the horse’s back using pressure mats, this direction of research is very promising [26, 27]. On the other hand, one of the GLM method imperfections is the nonsystematic coverage and poor presentation of image scales and directions. Therefore, GLCM and GLRLM are best suited for the detection of small lesions in low-resolution medial images [17]. Therefore, in future studies, the use of structural thermal image complexity analysis is preferred to the small size of the ROIs then to the whole body assessment.

## Conclusion

The textural analysis, including selected features of GLCM or GLRLM, seems to be promising tools in considering the quantitative assessment of thermographic images of horses’ thoracolumbar region. The GLCM and GLRLM analyses allowed the differentiation of horses subjected to a load of 10 and 15% of their body weights while horseback riding in contrast to the previously used SBT analysis methods. However, both types of analyzing methods, using SBT and GLM features, allow the differences to be shown between thermal images obtained before and after riding. We hope our findings will shed new light on the possibility of assessing the thermographic images of the horses’ back region, which may be helpful for veterinarians, trainers, and owners.

## Methods

### Animals

**Fig. 2:**
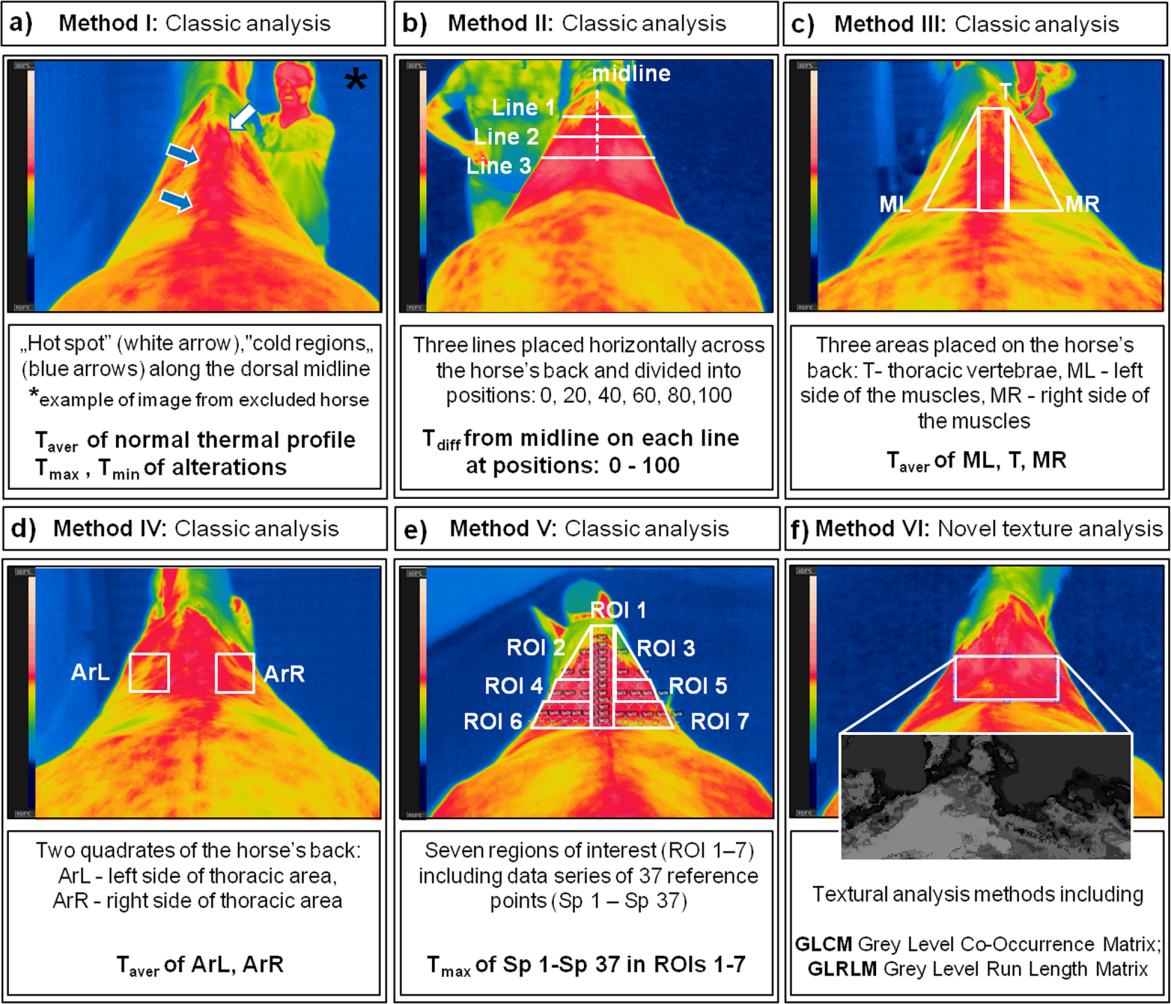
The methods chosen for classic and advanced analysis of the thoracic region of the horse’s back: **a** Schweinitz [2] and Fonseca et al. [6] method I; **b** Tunley and Henson [14] method II; **c** Soroko et al. [15] method III; **d** Pavelski et al. [16] method IV; **e** Masko et al. [12] method V; **f** novel texture analysis method

In the preliminary study participated eight Polish warmblood horses (eight geldings, mean age 8.2 ± 1.2 years, mean weight 540 kg). The horses were owned by the Warsaw School of Life Sciences and were in daily leisure use in the Didactic Stable of Horse Breeding Division. The ethics approval was deemed unnecessary according to regulations of the II Local Ethical Committee on Animal Testing in Warsaw and the National Ethical Committees on Animal Testing as well as according to Polish legal regulations (Ustawa z dnia 15 stycznia 2015 r. o ochronie zwierzat wykorzystywanych do celów naukowych lub edukacyjnych, Dz.U.2018.0.1207 (Resolution on the animals protection used for scientific and educational purposes), because all procedures in the study were non-invasive and did not cause distress and pain equal to or greater then a needlestick. To ensure that the horses were free from a preexisting inflammatory condition, clinical examinations were conducted before thermography according to the international veterinary standards [35, 36]. Basic clinical examinations included measurement of heart rate, mucous membranes (color and moisture), capillary refill time, dehydration (measured as the time it takes for a pinched skin fold over the point of the shoulder to flatten), and rectal temperature. Then, the thoracolumbar region was palpated carefully and the presence of tension in the muscles, lumps, abnormal hair wear and pain reaction were recorded. Only horses showing no clinical signs were included in the research. No horses were excluded during any of the clinical examinations. The thermographic examinations were carried out in accordance with the previously described protocol [3, 15]. Two horses with weak symptoms of back-pain or hypersensitivity and hot spots or cold regions visible on thermographic images along the dorsal midline were excluded at the stage of preliminary examination (Fig. 2a). Finally, six horses were qualified for this research. All six horses were clinically healthy, had no apparent back problems, and demonstrated a comparable conformation and athletic ability.

Six female riders (rider: A-E) with 4–5 years’ riding experience and the comparable rider’s training level participated in the study. Riders were members of the Animal Sciences Students Riding Association. The riders represented two different body weights: low body weight (LBW) - 50.3 ± 1.5 kg (rider: A, B, C) and high body weight (HBW) - 78.5 ± 1.8 kg (rider: D, E, F). Saddle weight was 4.1 to 4.4 kg respectively, hence body weight with saddle (kg)/% body weight of horse were 54.6 ± 1.4 kg/10.1 ± 0.003% for LBW riders and 82.7 ± 1.7 kg/15.3 ± 0.003% for HBW riders. During the experiment each horse worked under each rider, which allowed 36 combinations. For each horse, the 1 day break between the sessions was retained. The research was preceded by a six-month adaptation period in which the horses been housed and worked in the same environmental conditions including individual stalls, management, and feeding. At the end of the adaptation period, 1 week before the research, the saddles were fitted to each horse following Greve’s and Dyson’s (2015) protocol. The panels of the saddle, the type of flocking, and the balance of the saddle were determined. The saddle was considered not fit when evenness, lumps, depressions, lack of uniform thickness and softness, and lack of the left-right symmetry of the panels were recorded. The saddle was considered fit when in addition to the above conditions the lowest point of the seat of the saddle corresponded to the lowest point of the horse’s back [37].

### Thermographic data collection

The imaged area, thoracolumbar region, was brushed, and dirt and mud were removed 15 min before imaging [15]. Then the horses were led to an enclosed, indoor riding hall with constant environmental conditions. The hall was directly connected with the horses’ stable, therefore horses could participate in the research without having to contact the outside environment. The inside environment in the hall ensured to maintain the ambient temperature 20.2 ± 1.1 °C, protection from solar radiation and wind. Images were taken immediately before and after a training session, using a non-contact thermographic camera (FLIR Therma CAM E25, FLIR Systems Brasil, Brazil; emissivity (e) 0.99; temperature range between 26.4 and 36.8 °C. The camera was placed on a distance of approximately 1.2 m up from the imaging area, in front of the vertical axis marked base on the dorsal spinal processes L5. All thermographic images were obtained by the same researcher (MM). The same protocol was repeated for each horse in the following order: the first thermographic image was taken, the horse was saddled, a training session was performed, the horse was unsaddled, the second thermographic image was taken, and the horse was walked on the rope to complete rest. The training sessions lasted 50.0 ± 2.5 min, during with horses worked 10 min in both directions at walk (1.6 m/s), 15 min at trot (4.0 m/s), 5 min at walk (1.6 m/s), 10 min at canter (7.0 m/s) and in the end 10 min at walk (1.6 m/s).

### Thermographic data analysis

All thermographic images were analyzed independently using five simple analysis methods (I, II, III, IV, V) and the novel texture analysis method (VI).

The method I was used by Schweinitz [2] and Fonseca et al. [6] and based on the recognition of “hot spots” or “cold regions” (Fig. 2a). Direct marking and visualization of the exact locations of alterations in the thermal pattern were realized concerning the normal thermal profile. A temperature difference between antimeres within a range of 0.5 °C to 1 °C was considered normal. All alterations greater then 1 °C above the norm were defined as “hot spots”, whereas below as “cold regions”. In the method I the average temperature (T_aver_) of the normal thermal profile (NTP) as well as the maximal temperature (T_max_) and the minimal temperature (T_min_) of alterations - the “hot spot” or “cold region”, respectively, were determined.

In method II, used by Tunley and Henson [14], the thoracolumbar region of the back was imaged based on the analysis of the range temperatures measured along six horizontal lines. In this paper, we considered three lines from the thoracic region (Fig. 2b). The lines were placed horizontally across the images at specific anatomical sites: Line 1 at T9 (base of mane region), line 2 at T12 (base of withers region), line 3 at the same distance from line 2 as line 2 was from line 1 (T15 region). Each line was individually divided from the left into the positions 0, 20, 40, 60, 80 and 100. Then, the temperature of each horse at positions 0, 20, 40, 50, 60, 80 and 100 on each line were extrapolated from the graphs. The baseline being the midline measured as zero, and the other measurements the expected difference in °C from the midline. Measures were presented as the differences between temperature (T_diff_) at midline (0) and at subsequent positions: 0, 20, 40, 60, 80, 100.

The method III was described by Soroko et al. [15] and included a comparison of the average temperatures measured in five areas separated from the thoracolumbar region of the back. In this paper, we considered three areas from the thoracic region (Fig. 2c). The thoracic region of the back which includes axial skeleton form wither to lumbar vertebrae was divided into three areas: thoracic vertebrae (Th) and symmetric sides of thoracic vertebrae area: left side of the muscles (ML); right side of the muscles (MR) (Fig. 2c). Then, the average temperature of each area of each horse was obtained.

The method IV was conducted by Pavelski et al. [16] and measured the maximal, minimal and average temperatures in two selected quadrate of the thermographic image from the thoracic region of the horse’s back. The quadrates were the selected area of 20X20 pixels of the thoracic region (Ar), both sides left (ArL) and right (ArR). In this paper, we considered equivalents of those quadrates of back in the frontal plane (Fig. 2d). Then, the average temperature of both quadrates of each horse was received.

In method V, heat pattern of the thoracic region was evaluated using images 37 reference points (Sp 1–37) grouped into seven regions of interest (ROI 1–7) representing areas with an impact on specific groups of skeletal muscles (Fig. 2e). Then, the maximal temperature of each Sp were measured and included to data series of the specific region of interest (ROI): ROI 1 (Sp 1–13), ROI 2 (Sp 14–16), ROI 3 (Sp 17–19), ROI 4 (Sp 20–23), ROI 5 (Sp 24–27), ROI 6 (Sp 28–32) and ROI 7 (Sp 33–37).

The novel method VI, texture analysis methods can extract information from images using quantitatively analyzing gray levels distribution, pixel relationships, and co-occurrence of pixels spatially. In the first step, the color images were converted to a grayscale image. The texture analysis was conducted for ROI of the thoracic region (Fig. 2f). The mentioned features were computed using QMaZda software (a free-source website: http://www.eletel.p.lodz.pl/pms/SoftwareQmazda.html). Texture analysis using in this study includes Gray Level Co-Occurrence Matrix (GLCM) and Gray Level Run Length Matrix (GLRLM). Gray Level Co-Occurrence matrix is a mathematical tool based on the analysis of the spatial relationship of the intensity of two pixels in texture [17, 22, 38]. The distance between analyzed pixels was set to 5 in the vertical direction. In the particular software which was used 11 features of GLCM method are computed: angular second moment/energy (AngScMom), contrast (Contrast), correlation (Correlate), variance/sum of squares (SumOfSqs), inverse different moment/homogeneity (InvDefMom), sum average (SumAverg), sum variance (SumVarnc), sum entropy (SumEntrp), entropy (Entropy), difference variance (DifVarnc), difference entropy (DifEntrp). AngScMom and Entropy belong to measures of order. Energy measures homogeneity of texture, its high value indicates constant or repeatable brightness of pixels. Entropy measures disorder in the texture or complexity, its high value indicates a heterogeneous texture. The other measures include contrast measures and statistics describing a matrix such as correlation or variance. Contrast is called the sum of squares variance, its low value indicates a lack of difference between pixels. Homogeneity is measure inversely proportional to the contrast if the contrast decreases InvDefMom increases. Variance indicates the value dispersion relative to the average. Correlation shows the linear relationship between two neighboring pixels that are expressed by the regression equation. In addition to the parameters shown, there are variance and entropy calculated on the sum or the difference of adjacent pixels.

Gray Level Run Length Matrix is the second mathematical tool of the texture analysis, which aim is to find runs of consecutive pixels with the same gray level value in the given direction [22, 39]. In Mazda software was calculated 7 features of GLRLM method: short-run emphasis (SRE), long-run emphasis (LRE), gray level non-uniformity (GLN), run-length nonuniformity (RLN), a fraction of image in runs (Fraction), run-length nonuniformity moment (MRLN), gray level non-uniformity moment (MGLN). The fraction of the image in runs determines the percentage of the runs considered for matrix computing. The short-run and the long run emphasis show the proportion of runs of short length and long length occurring in the image.

### Statistical analysis

The data from each horse-rider combinations were presented in form of data series for each analysis methods, independently: for method I - T_aver_ of NTP, T_max_ of alterations, T_min_ of alterations; for method II - T_diff_ at position 0, T_diff_ at position 20, T_diff_ at position 40, T_diff_ at position 60, T_diff_ at position 80, T_diff_ at position 100; for method III - T_aver_ of ML, T_aver_ of T, T_aver_ of MR; for method IV - T_aver_ of ArL, T_aver_ of ArR; for method V - T_max_ in ROI 1, T_max_ in ROI 2, T_max_ in ROI 3, T_max_ in ROI 4, T_max_ in ROI 5, T_max_ in ROI 6, T_max_ in ROI 7, T_max_ of entire body; for method VI - GLCM features (Contrast, Correlate, InvDefMom, Entropy, DifVarnc, DifEntrp), and GLRLM features (RLN, GLN, LRE, SRE, Fraction, MRLN). All data series were tested independently for univariate marginal distributions using a univariate Kolmogorov–Smirnov test. The paired t-test (Gaussian distributed data) or Wilcoxon signed-rank test (non-Gaussian distributed data) was used to distinguish the thermographic images obtained before and after a training session. The unpaired t-test with Welch’s correction (Gaussian distributed data) or Mann Whitney test (non-Gaussian distributed data) was used to evidence the differences between two groups of riders: low body weight (LBW) and high body weight (HBW). All results were reported on the figures as mean ± SD. All statistical analysis was performed using GraphPad Prism6 software (GraphPad Software Inc., CA, USA), where the significance level was established as *p* < 0.05.

## Data Availability

All data generated or analyzed during this study are included in this published article. If any additional material used and/or analyzed during the current study is required, these are available from the corresponding author on reasonable request. The features of statistical texture analysis were computed using QMaZda software (version 19.02, available at http://www.eletel.p.lodz.pl/pms/SoftwareQmazda.html).

## Abbreviations

AngScMom: Angular second moment/energy
Ar: Thoracic region
ArL: Left side of the thoracic region
ArR: Right side of the thoracic region
Correlate: Correlation
DifEntrp: Difference entropy
DifVarnc: Difference variance
Fraction: A fraction of image in runs
GLCM: Gray Level Co-Occurrence Matrix
GLN: Gray level non-uniformity
GLRLM: Gray Level Run Length Matrix
HBW: High body weight
InvDefMom: Inverse different moment/homogeneity
LBW: Low body weight
LRE: Long-run emphasis
MGLN: Gray level non-uniformity moment
ML: Left side of the muscles
MR: Right side of the muscles
MRLN: Run-length nonuniformity moment
NTP: The normal thermal profile
RLN: Run-length nonuniformity
ROI: Region of interest
Sp: Reference points
SRE: Short-run emphasis
SumAverg: Sum average
SumEntrp: Sum entropy
SumOfSqs: Variance/sum of squares
SumVarnc: Sum variance
T_aver_: The average temperature
T_diff_: The differences between temperature
Th: Thoracic vertebrae
T_max_: The maximal temperature
T_min_: The minimal temperature

## References

[1] Turner TA (2001). Diagnostic thermography. Vet Clin North Am Equine Pract.

[2] von Schweinitz D (1999). Thermographic diagnostics in equine back pain. Vet Clin North Am Equine Pract.

[3] von Hoogmoed LM, Snyder JR, Allen AK (2000). Use of infrared thermography to detect performance-enhancing techniques in horses. Equine Vet Educ.

[4] Soroko M, Howell K (2018). Infrared thermography: current applications in equine medicine. J Equine Vet Sci.

[5] Ring EFJ, Thomas R, Howell K, Jones DP (2009). Sensors for medical thermography and infrared radiation measurements. Biomedical sensors.

[6] Fonseca BPA, Alves ALG, Nicoletti JLM (2006). Thermography and ultrasonography in back pain diagnosis of equine athletes. J Equine Vet Sci.

[7] Ciutacu O, Tanase A, Miclaus I (2006). Digital infrared thermography in assessing soft tissues injuries on sport equines. Bull Univ Agric Sci Vet Med Cluj Napoca.

[8] Kastberger G, Stachl R (2003). Infrared imaging technology and biological applications. Behav Res Methods Instrum Comput.

[9] Visser EK, Neijenhuis F, de Graaf-Roelfsema E (2014). Risk factors associated with health disorders in sport and leisure horses in the Netherlands. J Anim Sci.

[10] Haussler KK (1999). Back problems. Chiropractic evaluation and management. Vet Clin North Am Equine Pract.

[11] Haussler KK, Jeffcott LB (2014). Back and pelvis, section 2: musculoskeletal system. Equine sports medicine and surgery.

[12] Masko M, Krajewska A, Zdrojkowski L (2019). An application of temperature mapping of horse’s back for leisure horse-rider-matching. Anim Sci J.

[13] Janczarek I, Wilk I (2017). Leisure riding horses: research topics versus the needs of stakeholders. Anim Sci J.

[14] Tunley BV, Henson FM (2004). Reliability and repeatability of thermographic examination and the normal thermographic image of the thoracolumbar region in the horse. Equine Vet J.

[15] Soroko M, Jodkowska E, Zablocka M (2012). The use of thermography to evaluate back musculoskeletal responses of young racehorses to training. Thermol Int.

[16] Pavelski M, da Silva Basten IM, Busato E (2015). Infrared thermography evaluation from the back region of healthy horses in controlled temperature room. Cienc Rural.

[17] Depeursinge A, Al-Kadi OS, Mitchell JR (2017). Biomedical texture analysis: fundamentals, tools, and challenges.

[18] Honeycutt CE, Plotnick R (2008). Image analysis techniques and gray-level co-occurrence matrices (GLCM) for calculating bioturbation indices and characterizing biogenic sedimentary structures. Comput Geosci.

[19] Malegori C, Franzetti L, Guidetti R (2016). GLCM, an image analysis technique for early detection of biofilm. J Food Eng.

[20] Sohail ASM, Bhattacharya P, Mudur SP, et al. Local relative GLRLM-based texture feature extraction for classifying ultrasound medical images. In 2011 24th CCECE. IEEE. 2011;(May):001092–5. doi: 10.1109/CCECE.2011.6030630

[21] Zhang H, Hung CL, Min G (2019). GPU-accelerated GLRLM algorithm for feature extraction of MRI. Sci Rep.

[22] Girejko G, Borowska M, Szarmach J (2018). Statistical analysis of radiographic textures illustrating healing process after the guided bone regeneration surgery.

[23] Durgamahanthi V, Christaline JA, Edward AS (2021). GLCM and GLRLM based texture analysis: application to brain cancer diagnosis using histopathology images. Intelligent computing and applications.

[24] McKeever KH (2004). Body fluids and electrolytes: responses to exercise and training. Equine sports medicine and surgery.

[25] Hyyppa S, Poso A (2004). Metabolic diseases of athletic horses. Equine sports medicine and surgery.

[26] Pagan J, Hintz H (1986). Equine energetics. II. Energy expenditure in horses during submaximal exercise. J Anim Sci.

[27] Powell D, Bennett-Wimbush K, Peeples A (2008). Evaluation of indicators of weight-carrying ability of light riding horses. J Equine Vet Sci.

[28] Ille N, Aurich C, Erber R (2014). Physiological stress responses and horse rider interactions in horses ridden by male and female riders. Comp Exerc Physiol.

[29] Wilk I, Wnuk-Pawlak E, Janczarek I (2020). Distribution of superficial body temperature in horses ridden by two riders with varied body weights. Animals..

[30] Soroko, Zaborski D, Dudek K (2019). Evaluation of thermal pattern distributions in racehorse saddles using infrared thermography. PLoS One.

[31] Peham C, Kotschwar AB, Brokenhagen B (2010). A comparison of forces acting on the horse’s back and the stability of the rider’s seat in different positions at the trot. Vet J.

[32] Meschan EM, Peham C, Schobesberger H (2007). The influence of the width of the saddle tree on the forces and the pressure distribution under the saddle. Vet J.

[33] Belock B, Kaiser LJ, Lavagnino M (2012). Comparison of pressure distribution under a conventional saddle and a treeless saddle at sitting trot. Vet J.

[34] Peham C, Licka T, Girtler D (2001). Hindlimb lameness: clinical judgement versus computerised symmetry measurement. Vet Rec.

[35] Martin BB, Klide AM (1999). Physical examination of horses with back pain. Vet Clin North Am Equine Pract.

[36] Purohit R (2009). Standards for thermal imaging in veterinary medicine. 11th European Congress of Thermology. Thermol Int.

[37] Greve L, Dyson S (2015). Saddle fit and management: an investigation of the association with equine thoracolumbar asymmetries, horse and rider health. Equine Vet J.

[38] Haralick MR (1979). Statistical and structural approaches to texture. Proc IEEE.

[39] Galloway MM. Texture classification using gray level run length. Comput Graph Image Process. 1975. 10.1016/S0146-664X(75)80008-6.

